# Circulating Tumor Cells in Renal Cell Carcinoma: Recent Findings and Future Challenges

**DOI:** 10.3389/fonc.2019.00228

**Published:** 2019-04-05

**Authors:** Matteo Santoni, Alessia Cimadamore, Liang Cheng, Antonio Lopez-Beltran, Nicola Battelli, Francesco Massari, Marina Scarpelli, Andrea Benedetto Galosi, Sergio Bracarda, Rodolfo Montironi

**Affiliations:** ^1^Oncology Unit, Macerata Hospital, Macerata, Italy; ^2^Section of Pathological Anatomy, School of Medicine, United Hospitals, Polytechnic University of the Marche Region, Ancona, Italy; ^3^Department of Pathology and Laboratory Medicine, Indiana University School of Medicine, Indianapolis, IN, United States; ^4^Department of Pathology and Surgery, Faculty of Medicine, Córdoba, Spain; ^5^Division of Oncology, S. Orsola-Malpighi Hospital, Bologna, Italy; ^6^Department of Urology, School of Medicine, United Hospitals, Marche Polytechnic University, Ancona, Italy; ^7^Medical Oncology, Department of Oncology, Azienda Ospedaliera S. Maria, Terni, Italy

**Keywords:** circulating tumor cells, diagnosis, isolation techniques, prognosis, renal cell carcinoma, circulating tumor microemboli

## Introduction

Renal cell carcinoma (RCC) is the most common tumor of the kidney. After diagnosis, 20–30% of patients will relapse, with a high probability of death from cancer-related causes. The development of non-invasive biomarkers will allow the identification of patients with a high risk of recurrence after radical or partial nephrectomy and will improve the assessment of tumor response to targeted therapy or immunotherapy. The search for non-invasive diagnostic techniques represents one of the most difficult challenges for cancer researchers. The contemporary scenario includes a variety of strategies that share the aim of maximally reducing the impact of the diagnosis on patients' quality of life (QoL). In this context, liquid biopsy offers a promising perspective for cancer diagnosis and monitoring, with several advantages compared to traditional diagnostic procedures (1). Indeed, it can be more frequently performed and allows for better tracking of tumors and mutations over a period of time (1). Moreover, it has been showed that genomic profiles of liquid biopsies can match very closely with the corresponding tumors (2).

The liquid biopsy of circulating tumor cells (CTCs), which belong to the larger family of circulating rare cells (CRC), has been validated and approved by the US Food and Drug Administration (FDA) as a useful prognostic tool in a variety of cancer types (3). This is based not only on the ability of CTCs to be a mirror of tumor heterogeneity but also on the possibility to combine the genetic and transcriptomic status of single CTCs (4) with epigenome analyses (5). Although CTCs have received great attention based on their potential in evaluating the status of localized and metastatic diseases, their clinical implementation is not yet widespread.

In the last decade, several studies have investigated the clinical and pathological significance of CTC numbers and characteristics in patients with urogenital cancers (6, 7). In view of the increasing number of dedicated clinical trials, genitourinary tumors represent the next urgent field of application of molecular diagnostics and drug discovery after gastro-intestinal and thoracic oncology. Among these tumors, the absence of reliable predictive biomarkers in RCC prevents the proper selection of patients who will benefit from any one of the three main drug categories approved for treating this disease: (1) anti-vascular endothelial growth factor (VEGF) monoclonal antibodies (i.e., bevacizumab) or tyrosine kinase inhibitors (i.e., sunitinib, sorafenib, pazopanib, axitinib, cabozantinib, tivozanib, and lenvatinib); (2) immune checkpoint inhibitors [i.e., anti-programmed death(PD)-1 Nivolumab alone or in combination with anti-cytotoxic T-lymphocyte antigen (CTLA)-4]; and (3) Mammalian target of rapamycin (mTOR) inhibitors (i.e., everolimus and temsirolimus). In this manuscript we describe the emerging data on the role of CTCs in the diagnosis and treatment of RCC, focusing on their future applicability in daily clinical practice.

## Intra- and Inter-tumor Heterogeneity in RCC

Intratumoral heterogeneity—in terms of somatic mutations, chromosome aberrations, and tumor gene expression—is a characteristic feature of clear cell RCC (8–11). These alterations are primarily centered around the *Von Hippel–Lindau (VHL)* gene and include LOH at 3p and epigenetic silencing. *VHL* inactivation is a crucial event in the majority of clear cell RCC and represents the only ubiquitous event, in contrast from the other genetic and/or epigenetic aberrations reported which are just subclonal (12–14). In this setting, Xu et al. firstly revealed by single-cell exome sequencing in a single patient that <30% of gene alterations are common to multiple cells within tumor tissue, whilst the majority are only cell-specific (15). In the same view, activated drug target pathways have been shown to be considerably variable within the same tumor and between primary RCC and lung metastases (16).

The rate of patients with late-relapsing disease (>5 y after radical or partial nephrectomy) (17–19) and the common intravenous tumor embolization (20) are just two of a series of clear signs suggesting that CTCs may provide fundamental information to optimize RCC diagnosis and evaluate tumor response to therapy and progression.

## CTC Isolation and Characterization in RCC

The collection, identification, enrichment, and analysis of CTC require the use of different methods, including the following: (1) Epithelial or non-epithelial marker-dependent isolation; (2) RT-PCR-based methods; (3) and morphological- and cell size-based detection (21).

**Figure 1:**
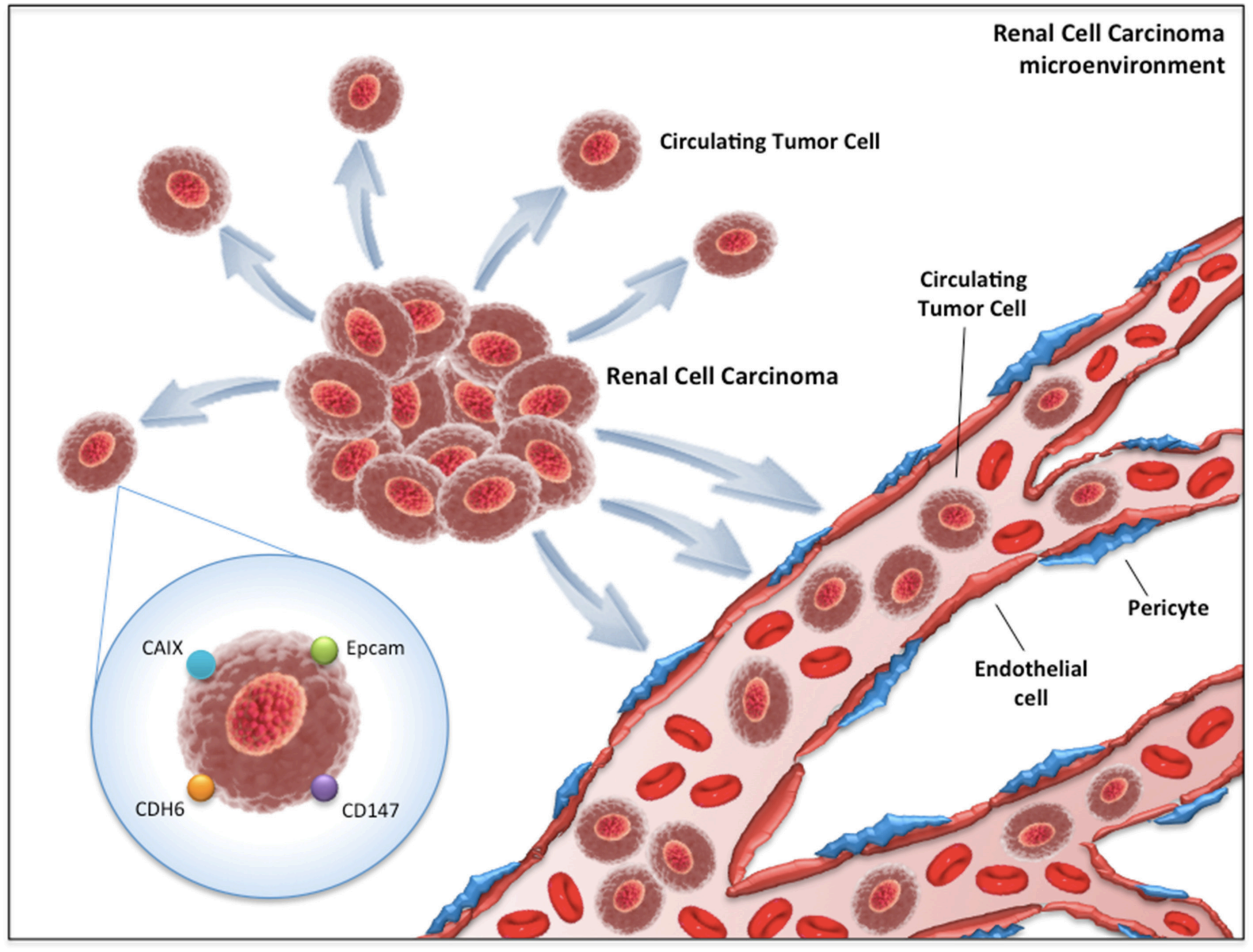
Circulating tumor cells (CTCs) in renal cell carcinoma microenvironment. CAIX, Carbonic anhydrase 9; CDH6, Cadherin-6; EpCAM, Epithelial cell adhesion molecule.

The first method consists of the detection of CTCs through epithelial markers, such as the epithelial cell adhesion molecule (EpCAM, Figure 1). EpCAM is a transmembrane glycoprotein involved in cell signaling, migration, proliferation, and differentiation (22). The number of CTCs that can be isolated by EpCAM is usually low (23). This is based on the biological behavior of clear cell RCC, which often transdifferentiates through a process named “epithelial-to-mesenchymal transition (EMT),” a morphological transformation that is phenotypic of RCC cells (24, 25) and that leads to the loss of their epithelial antigens and the acquisition of mesenchymal features (i.e., vimentin expression). Detecting EMT markers on CTCs provides fundamental information on the status of the disease, considering the straight association between EMT and the prognosis of RCC patients (24) as well as its role in the acquisition of invasive properties and resistance to anti-VEGF TKIs (24). More recently, antibodies directed against membrane carbonic anhydrase 9 (CA9/CAIX) and CD147 [a widely expressed membrane glycoprotein involved in matrix metalloproteinase induction, cell adhesion and T cell activation (26)] have been developed to increase the number of selected CTCs in RCC patients (Figure 1). Indeed, Liu et al. reported that while EpCAM was found only in about 18% of clear cell RCC tumors, CAIX, and CD147 were present in more than 97% of samples (27).

The second method is based on the RT-PCR approach. The three main targets of this technique are *CAIX, VHL* and *Cadherin-6 (CDH-6)* (Figure 1). *VHL* gene alterations detected in tumor samples have reported concordance with those identified on peripheral blood in about 75% of cases (28). On the other hand, *CDH-6* gene expression by RT-PCR has been observed in 45% of clear cell RCC blood samples (29).

The third method is built upon size-based blood filtration combined with morphological and genetic analyses. The need to associate different techniques derives from the evidence that cytomorphological classification alone is not sufficient to detect CTCs in RCC patients (30). Interestingly, the use of these combined methods has enabled the detection of the presence of circulating clusters with a core of cancer cells surrounded by an external coating of endothelial cells (31).

The survival mechanisms underlying the circulation and migration of CTCs depend on multiple factors. Their biological characteristics, genetic alterations, epithelial mesenchymal transition, and cancer stem cell properties are internal factors that influence their survival. Great importance is now also being attributed to the external factors in the bloodstream microenvironment, consisting of platelets, immune cells, cytokines, and circulating tumor microemboli (CTM) (32).

**Figure 2:**
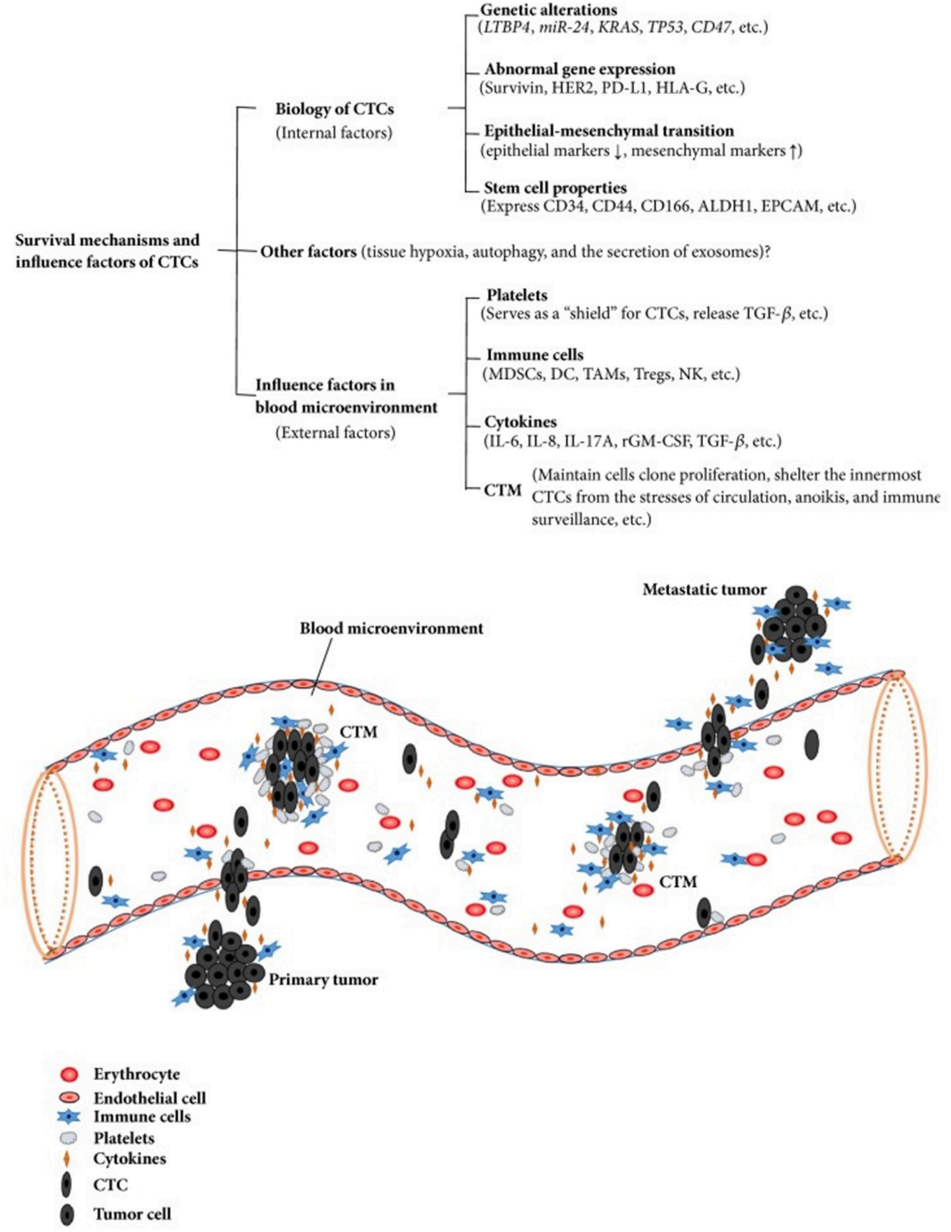
Survival mechanisms and influence factors of CTCs. Copyright © 2018 Wang et al. (32). This is an open access article distributed under the Creative Commons Attribution License, which permits unrestricted use, distribution, and reproduction in any medium, provided the original work is properly cited.

CTM are composed by cell clusters, from two to more than 50 CTCs, together with leukocytes, cancer-associated fibroblasts, endothelial cells, and platelets (33). CTM demonstrated high metastatic potential by inhibiting apoptosis, promoting cell clonal proliferation, conferring resistance to shear stress, and protecting the innermost cells from immune surveillance from the identification of NK cells and their cytolytic activity (34). Testing the expression of Ki-67 on CTCs and CTMs has revealed a sharp contrast between CTCs (Ki-67 positive) and CTMs (all negative for Ki-67), even in patients with Ki67 (+) CTCs (35). This gives rise to the hypothesis that CTMs, in comparison to CTCs, may have the capability to remain inactive and “dormant” for long time periods, inhibiting apoptosis and cell destruction in the bloodstream. They may also have the capacity to confer resistance to cytotoxic drugs for cells within CTM, as observed by Hou et al. in patients with non-small–cell lung cancer (36). Colorectal cancer patients with CTMs in their blood have a shorter survival period than patients with only CTCs detected (37). Similar results were obtained in blood samples from gastric cancer patients, in which the presence of CTMs was an independent predictor of shorter PFS and OS in stage IV patients in multivariate analysis (38). Detection of CTM has emerged as a valuable tool to improve the prognostic significance of liquid biopsy (39) (Figure 2). Comparing the epigenomes of CTC clusters and single CTCs, Gkountela et al. demonstrated that CTC clusters are enriched with binding sites for several key transcription factors (TF)—such as OCT4, NANOG, and SOX2—that confer to clusters stem cell features, whereas single CTC have enrichment in different sets of TF. Moreover, stem cell TF binding is lost when clusters are dissociated into single cells via Na+/K+ ATPase inhibitors such as digitoxin. In xenograft mouse models generated using *ex vivo* expanded CTC lines, administration of digitoxin significantly inhibited the capacity of CTC clusters to generate metastases (40, 41). These results open an area of new research and new therapeutic targets.

## New Methods of Detection

New methods of isolation and analysis such as subtraction enrichment (SE) combined with immunostaining-fluorescence *in situ* hybridization (iFISH) are being developed to better characterize CTCs. With this method, independent of cell size variation, and free of hypotonic damage as well as anti-EpCAM perturbing, it is possible to karyotype chromosome ploidy of CTCs and phenotype multi-protein expression. Among different cancers, an aneuploid chromosome 8 (tetraploid or polyploid) identified a positive CTC (42–44). This allows for efficient enrichment, identification, and characterization of both large and small size CTCs as well as CTM in various biofluid samples including cerebrospinal fluid (CSF). Unlike conventional methodologies, SE-iFISH enables the characterization of different heterogeneous CTC subtypes classified by both chromosome ploidy and the expression of biomarkers (44). Broncy et al. recently applied single-cell genetic analysis after isolation by SizE of Tumor/Trophoblastic cells (ISET®) in order to assess the specificity and sensitivity of cytopathology. They performed single cell analysis targeted to VHL mutations in all the 205 CRCs identified by cytopathology in the blood of 29 ccRCC patients after ISET filtration. They found a complete (100%) specificity of the cytopathological approach in the identification of circulating cancer cells (CCC) with a low sensitivity (35%) compared to genetic analysis (72%) (45, 46).

A novel straight microfluidic chip technology to focus and capture CTCs has been applied in head and neck cancer patients. The microchip is designed based on inertial migration of cells in a straight microchannel and allow to isolate single CTCs, CTCs clusters, and CTM by a size-based method, with high recovery efficiencies and low background cell contamination (47, 48).

## Future Perspectives

In future years, technical advances should aim to isolate a greater number of CTCs in metastatic patients than in patients with localized disease and to find the same mutations present in the correspondent histologic sample or, especially, in the metastatic cohort. In our opinion, this could be realized by improving the connection between cytomorphological and genetic analyses, thus overcoming the limits of present techniques, which can be challenging and time-consuming. Indeed, the specificity of CAIX and CDH-6 for CTCs in RCC patients is poor and could be increased only by the design of studies focused on comparing CTCs isolated from patients with clear cell and from patients with benign kidney diseases or healthy volunteers.

We hope that in future years whole genome, transcriptome, and proteome analyses of single cells could lead to an increase in our knowledge of tumor heterogeneity and acquired drug resistance. In the localized RCC setting, CTCs could have potential as a surveillance biomarker for disease recurrence. Earlier detection of metastatic RCC, prior to the onset of symptoms, may lead to improved clinical outcomes. In patients with metastatic disease, CTC analysis could be used to select patients for biomarker-guided clinical trials. As in colorectal cancers, the mutational profile of metastatic RCC could evolve after treatment progression, developing an acquired resistance to therapy potentially investigable in a non-invasive way with CTCs. Moreover, the introduction of data on CTCs within the TNM classification represents another step forward on the route of personalized medicine for RCC patients.

## Conclusions

RCC may benefit from the development of non-invasive and reliable biomarkers, enabling early and timely personalized treatment changes. The introduction of CTC analysis within daily clinical practice for patients with RCC seems still far at the moment. However, the advances obtained in the last 5 years in isolating and analyzing CTCs bring optimism about the future therapeutic landscape in RCC patients.

## Author Contributions

RM and MSc: conception and design; MSa, AC, and FM: drafting the manuscript; NB and ABG: review of the literature; LC, SB, and AL-B: critical revision of the manuscript.

### Conflict of Interest Statement

The authors declare that the research was conducted in the absence of any commercial or financial relationships that could be construed as a potential conflict of interest.

## References

[1] Crowley E Di Nicolantonio F Loupakis F Bardelli A. Liquid biopsy: monitoring cancer-genetics in the blood. Nat Rev Clin Oncol. (2013) 10:472–84. 10.1038/nrclinonc.2013.110 23836314

[2] Gingras I Salgado R Ignatiadis M Liquid biopsy: will it be the “magic tool” for monitoring response of solid tumors to anticancer therapies? Curr Opin Oncol. (2015) 27:560–7. 10.1097/CCO.0000000000000223 26335664

[3] Karachaliou N Mayo-de-Las-Casas C Molina-Vila MA Rosell R. Real-time liquid biopsies become a reality in cancer treatment. Ann Transl Med. (2015) 3:36. 10.3978/j.issn.2305-5839.2015.01.16 25815297 PMC4356857

[4] Klein CA Seidl S Petat-Dutter K Offner S Geigl JB Schmidt-Kittler O. Combined transcriptome and genome analysis of single micrometastatic cells. Nat. Biotechnol. (2002) 20:387–92. 10.1038/nbt0402-387 11923846

[5] Zhao C Hu S Huo X Zhang Y. Dr.seq2: a quality control and analysis pipeline for parallel single cell transcriptome and epigenome data. PLoS ONE. (2017) 12:e0180583. 10.1371/journal.pone.0180583 28671995 PMC5495495

[6] Montironi R Lopez-Beltran A Cheng L Cimadamore A Zizzi A Galosi A EAU Section of Uropathology (ESUP): uropathologists are fast moving forward to include blood (liquid) biopsy in their routine armamentarium. Eur Urol Today. (2018) 2018:26–27.

[7] Bergerot PG Hahn AW Bergerot CD Jones J Pal SK. The role of circulating tumor DNA in renal cell carcinoma. Curr Treat Options Oncol. (2018) 19:10. 10.1007/s11864-018-0530-4 29464405

[8] Santoni M Santini D Massari F Conti A Iacovelli R Burattini L. Heterogeneous drug target expression as possible basis for different clinical and radiological response to the treatment of primary and metastatic renal cell carcinoma: suggestions from bench to bedside. Cancer Metastasis Rev. (2014) 33:321–31. 10.1007/s10555-013-9453-5 24337954

[9] Piva F Santoni M Matrana MR Satti S Giulietti M Occhipinti G. BAP1, PBRM1 and SETD2 in clear cell renal cell carcinoma: molecular diagnostics and possible targets for personalized therapies. Expert Rev Mol Diagn. (2015) 15:1201–10. 10.1586/14737159.2015.1068122 26166446

[10] Massari F Ciccarese C Santoni M Brunelli M Piva F Modena A. Metabolic alterations in renal cell carcinoma. Cancer Treat Rev. (2015) 41:767–76. 10.1016/j.ctrv.2015.07.002 26169313

[11] Piva F Giulietti M Occhipinti G Santoni M Massari F Sotte V. Computational analysis of the mutations in BAP1, PBRM1 and SETD2 genes reveals the impaired molecular processes in renal cell carcinoma. Oncotarget. (2015) 6:32161–8. 10.18632/oncotarget.5147 26452128 PMC4741666

[12] Gerstung M Beisel C Rechsteiner M Wild P Schraml P Moch H. Reliable detection of subclonal single-nucleotide variants in tumour cell populations. Nat. Commun. (2012) 3:811. 10.1038/ncomms1814 22549840

[13] Gerlinger M Rowan AJ Horswell S Math M Larkin J Endesfelder D. Intratumor heterogeneity and branched evolution revealed by multiregion sequencing. N Engl J Med. (2012) 366:883–92. 10.1056/NEJMoa1113205 22397650 PMC4878653

[14] Gerlinger M Horswell S Larkin J Rowan AJ Salm MP Varela I. Genomic architecture and evolution of clear cell renal cell carcinomas defined by multiregion sequencing. Nat Genet. (2014) 46:225–33. 10.1038/ng.2891 24487277 PMC4636053

[15] Xu X Hou Y Yin X Bao L Tang A Song L. Single-cell exome sequencing reveals single-nucleotide mutation characteristics of a kidney tumor. Cell. (2012) 148:886–95. 10.1016/j.cell.2012.02.025 22385958 PMC7458411

[16] Kim KT Lee HW Lee HO Song HJ Jeong da E Shin S. Application of single-cell RNA sequencing in optimizing a combinatorial therapeutic strategy in metastatic renal cell carcinoma. Genome Biol. (2016) 17:80. 10.1186/s13059-016-0945-9 27139883 PMC4852434

[17] Santoni M Conti A Porta C Procopio G Sternberg CN Basso U. Sunitinib, pazopanib or sorafenib for the treatment of patients with late-relapsing (>5 years) metastatic renal cell carcinoma. J Urol. (2015) 193:41–7. 10.1016/j.juro.2014.07.011 25046616

[18] Santoni M Buti S Conti A Porta C Procopio G Sternberg CN. Prognostic significance of host immune status in patients with late relapsing renal cell carcinoma treated with targeted therapy. Targeted Oncol. (2015) 10:517–22. 10.1007/s11523-014-0356-3 25559290

[19] Santoni M Conti A Procopio G Porta C Ibrahim T Barni S. Bone metastases in patients with metastatic renal cell carcinoma: are they always associated with poor prognosis? J Exp Clin Cancer Res. (2015) 34:10. 10.1186/s13046-015-0122-0 25651794 PMC4328067

[20] Gu L Li H Wang Z Wang B Huang Q Lyu X. A systematic review and meta-analysis of clinicopathologic factors linked to oncologic outcomes for renal cell carcinoma with tumor thrombus treated by radical nephrectomy with thrombectomy. Cancer Treat Rev. (2018) 69:112–20. 10.1016/j.ctrv.2018.06.014 29960124

[21] van der Toom EE Verdone JE Gorin MA Pienta KJ. Technical challenges in the isolation and analysis of circulating tumor cells. Oncotarget. (2016) 7:62754–66. 10.18632/oncotarget.11191 27517159 PMC5308763

[22] Maetzel D Denzel S Mack B Canis M Went P Benk M. Nuclear signalling by tumour-associated antigen EpCAM. Nat Cell Biol. (2009) 11:162–71. 10.1038/ncb1824 19136966

[23] Gradilone A Iacovelli R Cortesi E Raimondi C Gianni W Nicolazzo C. Circulating tumor cells and “suspicious objects” evaluated through CellSearch® in metastatic renal cell carcinoma. Anticancer Res. (2011) 31:4219–21. 22199284

[24] Montironi R Santoni M Scarpelli M Piva F Lopez-Beltran A Cheng L. Re: epithelial-to-mesenchymal transition in renal neoplasms. Eur Urol. (2015) 68:736–7. 10.1016/j.eururo.2015.06.031 26334119

[25] Piva F Giulietti M Santoni M Occhipinti G Scarpelli M Lopez-Beltran A. Epithelial to mesenchymal transition in renal cell carcinoma: implications for cancer therapy. Mol Diagn Ther. (2016) 20:111–7. 10.1007/s40291-016-0192-5 26940073

[26] Hanna SM Kirk P Holt OJ Puklavec MJ Brown MH Barclay AN. A novel form of the membrane protein CD147 that contains an extra Ig-like domain and interacts homophilically. BMC Biochem. (2003) 4:17. 10.1186/1471-2091-4-17 14606962 PMC280649

[27] Liu S Tian Z Zhang L Hou S Hu S Wu J. Combined cell surface carbonic anhydrase 9 and CD147 antigens enable high-efficiency capture of circulating tumor cells in clear cell renal cell carcinoma patients. Oncotarget. (2016) 7:59877–91. 10.18632/oncotarget.10979 27494883 PMC5312355

[28] Ashida S Okuda H Chikazawa M Tanimura M Sugita O Yamamoto Y. Detection of circulating cancer cells with von Hippel-Lindau gene mutation in peripheral blood of patients with renal cell carcinoma. Clin Cancer Res. (2000) 6:3817–22. 11051223

[29] Li G Passebosc-Faure K Gentil-Perret A Lambert C Genin C Tostain J. Cadherin-6 gene expression in conventional renal cell carcinoma: a useful marker to detect circulating tumor cells. Anticancer Res. (2005) 25:377–81. 15816561

[30] El-Heliebi A Kroneis T Zöhrer E Haybaeck J Fischereder K Kampel-Kettner K. Are morphological criteria sufficient for the identification of circulating tumor cells in renal cancer? J. Transl. Med. (2013) 11:214. 10.1186/1479-5876-11-214 24044779 PMC3848446

[31] Kats-Ugurlu G Roodink I de Weijert M Tiemessen D Maass C Verrijp K. Circulating tumour tissue fragments in patients with pulmonary metastasis of clear cell renal cell carcinoma. J Pathol. (2009) 219:287–93. 10.1002/path.2613 19731255

[32] Wang W-C Zhang X-F Peng J Li X-F Wang A-L Bie Y-Q. Survival mechanisms and influence factors of circulating tumor cells. Biomed Res Int. (2018) 2018:6304701. 10.1155/2018/6304701 30515411 PMC6236925

[33] Krebs MG Metcalf RL Carter L Brady G Blackhall FH Dive C. Molecular analysis of circulating tumour cells—biology and biomarkers. Nat Rev Clin Oncol. (2014) 11:129–44. 10.1038/nrclinonc.2013.253 24445517

[34] Palumbo JS Talmage KE Massari JV La Jeunesse CM Flick MJ Kombrinck KW. Platelets and fibrin(ogen) increase metastatic potential by impeding natural killer cell-mediated elimination of tumor cells. Blood. (2005) 105:178–85. 10.1182/blood-2004-06-2272 15367435

[35] Li J Sharkey CC Wun B Liesveld JL King MR. Genetic engineering of platelets to neutralize circulating tumor cells. J Control Release. (2016) 228:38–47. 10.1016/j.jconrel.2016.02.036 26921521 PMC4828270

[36] Hou JM Krebs MG Lancashire L Sloane R Backen A Swain RK. Clinical significance and molecular characteristics of circulating tumor cells and circulating tumor microemboli in patients with small-cell lung cancer. J Clin Oncol. (2012) 30:525–32. 10.1200/JCO.2010.33.3716 22253462

[37] Zhang D Zhao L Zhou P Ma H Huang F Jin M. Circulating tumor microemboli (CTM) and vimentin+ circulating tumor cells (CTCs) detected by a size-based platform predict worse prognosis in advanced colorectal cancer patients during chemotherapy. Cancer Cell Int. (2017) 17:6. 10.1186/s12935-016-0373-7 28070168 PMC5217234

[38] Zheng X Fan L Zhou P Ma H Huang S Yu D. Detection of circulating tumor cells and circulating tumor microemboli in gastric cancer. Transl Oncol. (2017) 10:431–41. 10.1016/j.tranon.2017.02.007 28448959 PMC5406582

[39] Umer M Vaidyanathan R Nguyen NT Shiddiky MJA. Circulating tumor microemboli: Progress in molecular understanding and enrichment technologies. Biotechnol Adv. (2018) 36:1367–89. 10.1016/j.biotechadv.2018.05.002 29753882

[40] Gkountela S Castro-Giner F Szczerba BM Vetter M Landin J Scherrer R. Circulating tumor cell clustering shapes dna methylation to enable metastasis seeding. Cell. (2019) 176:98–112.e14. 10.1016/j.cell.2018.11.046 30633912 PMC6363966

[41] Yu M. Metastasis stemming from circulating tumor cell clusters. Trends Cell Biol. (2019) 29:P275–6. 10.1016/j.tcb.2019.02.001 30799250 PMC7771971

[42] Lin PP. Aneuploid CTC and CEC. Diagnostics. (2018) 8:E26. 10.3390/diagnostics8020026 29670052 PMC6023477

[43] Ye Z Ding Y Chen Z Li Z Ma S Xu Z. Detecting and phenotyping of aneuploid circulating tumor cells in patients with various malignancies. Cancer Biol Ther. (2018) 20:546–51. 10.1080/15384047.2018.1538000 30572767 PMC6422472

[44] Ge F Zhang H Wang DD Li L Lin PP. Enhanced detection and comprehensive *in situ* phenotypic characterization of circulating and disseminated heteroploid epithelial and glioma tumor cells. Oncotarget. (2015) 6:27049–64. 10.18632/oncotarget.4819 26267323 PMC4694973

[45] Broncy L Paterlini-Bréchot P. Circulating tumor cells for the management of renal cell carcinoma. Diagnostics. (2018) 8:63. 10.3390/diagnostics8030063 30177639 PMC6164661

[46] Broncy L Njima BB Méjean A Béroud C Romdhane KB Ilie M. Single-cell genetic analysis validates cytopathological identification of circulating cancer cells in patients with clear cell renal cell carcinoma. Oncotarget. (2018) 9:20058–74. 10.18632/oncotarget.25102 29732003 PMC5929446

[47] Kulasinghe A Zhou J Kenny L Papautsky I Punyadeera C. Capture of circulating tumour cell clusters using straight microfluidic chips. Cancers. (2019) 11:E89. 10.3390/cancers11010089 30646614 PMC6356955

[48] Zhou J Kulasinghe A Bogseth A O'Byrne K Punyadeera C Papautsky I Isolation of circulating tumor cells in non-small-cell-lung cancer patients using a multi-flow microfluidic channel. Nat Microsyst Nanotechnol. (2018) 5:8 10.1038/s41378-019-0045-6 PMC6387977 31057935

